# Specific Increase in MDR1 Mediated Drug-Efflux in Human Brain Endothelial Cells following Co-Exposure to HIV-1 and Saquinavir

**DOI:** 10.1371/journal.pone.0075374

**Published:** 2013-10-03

**Authors:** Upal Roy, Christine Bulot, Kerstin Honer zu Bentrup, Debasis Mondal

**Affiliations:** 1 Department of Pharmacology, Tulane University Health Sciences Center, New Orleans, Louisiana, United States of America; 2 Department of Microbiology and Immunology, Tulane University Health Sciences Center, New Orleans, Louisiana, United States of America; University of Cambridge, United Kingdom

## Abstract

Persistence of HIV-1 reservoirs within the Central Nervous System (CNS) remains a significant challenge to the efficacy of potent anti-HIV-1 drugs. The primary human Brain Microvascular Endothelial Cells (HBMVEC) constitutes the Blood Brain Barrier (BBB) which interferes with anti-HIV drug delivery into the CNS. The ATP binding cassette (ABC) transporters expressed on HBMVEC can efflux HIV-1 protease inhibitors (HPI), enabling the persistence of HIV-1 in CNS. Constitutive low level expression of several ABC-transporters, such as MDR1 (a.k.a. P-gp) and MRPs are documented in HBMVEC. Although it is recognized that inflammatory cytokines and exposure to xenobiotic drug substrates (e.g HPI) can augment the expression of these transporters, it is not known whether concomitant exposure to virus and anti-retroviral drugs can increase drug-efflux functions in HBMVEC. Our *in vitro* studies showed that exposure of HBMVEC to HIV-1 significantly up-regulates both MDR1 gene expression and protein levels; however, no significant increases in either MRP-1 or MRP-2 were observed. Furthermore, calcein-AM dye-efflux assays using HBMVEC showed that, compared to virus exposure alone, the MDR1 mediated drug-efflux function was significantly induced following concomitant exposure to both HIV-1 and saquinavir (SQV). This increase in MDR1 mediated drug-efflux was further substantiated via increased intracellular retention of radiolabeled [^3^H-] SQV. The crucial role of MDR1 in ^3^H-SQV efflux from HBMVEC was further confirmed by using both a MDR1 specific blocker (PSC-833) and MDR1 specific siRNAs. Therefore, MDR1 specific drug-efflux function increases in HBMVEC following co-exposure to HIV-1 and SQV which can reduce the penetration of HPIs into the infected brain reservoirs of HIV-1. A targeted suppression of MDR1 in the BBB may thus provide a novel strategy to suppress residual viral replication in the CNS, by augmenting the therapeutic efficacy of HAART drugs.

## Introduction

Being a lentivirus, HIV-1 infection shows a long latency period and slow disease progression resulting in severe immune deficiencies and ultimately AIDS. HIV-1 primarily infects CD4+ T-lymphocytes, but long-term persistence of the virus occurs in monocytes and macrophages, e.g. in microglial cells within the CNS. Thus, the sequestered sites within the brain can act as anatomical reservoirs for HIV-1 which prevents complete eradication of the virus despite long-term anti-retroviral therapy [1]–[3]. The Highly Active Antiretroviral Therapy (HAART) which is highly effective in suppressing serum viral load, cannot effectively suppress productive infection in these reservoirs. In the brain, one of the strategies via which HIV-1 can evade HAART efficacy is due to the presence of drug-efflux transporters on the BBB endothelial cells (EC) [4]. All of the currently available HIV-1 protease inhibitors (HPI) are known to be substrates of different ABC-transporters, and are actively effluxed from the BBB via both MDR1 (P-gp) and the multidrug resistance associated proteins (MRP-1 and MRP-2) [5], [6]. Although several competitive inhibitors of these transporters are being tested as an approach to increase HPI levels within the brain, their generalized inhibition has not been a safe and feasible approach since these drug-efflux pumps play crucial physiologic roles in protecting the CNS and other organs from toxic xenobiotics [7]. Therefore, a clear understanding of the molecular mechanism of ABC-transporter expression in HIV-infected brain microenvironments may provide novel strategies towards alternative treatment options such as their specific inhibition via gene therapy approaches.

Our previous studies demonstrated that these transporters are present in the ECs of different tissues including the BBB, and are responsible for the efflux of different anti-HIV-1 medications, especially the HPIs and the Nucleoside Reverse Transcriptase Inhibitors (NRTIs) [8]. Previous publications have also suggested that the HIV-1 transactivator (Tat) protein and/or HIV-1 induced inflammatory cytokines (e.g. TNF-α IL-1β etc.) can enhance the expression of different ABC transporters [9]–[11]. Nonetheless, very little is known about the specific drug transporters involved in drug-efflux from the BBB in HIV-1 infected microenvironments and their effector mechanisms which ultimately results in lower HAART levels within the CNS.

In the current study, an *in vitro* model of the BBB was adopted by utilizing primary HBMVEC, where we have documented changes in expression of both MDR1 and MRPs in cells exposed to HIV-1 and/or saquinavir (SQV) [12]. Furthermore, using different ABC-transporter inhibitors, e.g. verapamil, MK-571 and PSC-833, we have observed significant changes in the efflux function of both MDR1 and MRPs. We also demonstrated the dominant role of MDR1 in saquinavir efflux from HIV-1-exposed HBMVEC. Our findings implicate the therapeutic potential of specifically suppressing MDR1 expression to increase HAART entry into HIV-1-infected regions of the brain.

## Materials and Methods

### Reagent

The fluorescent dye Calcein acetoxy-methyl ester (Calcein-AM) was obtained from Molecular Probes (Eugene, OR). The dual inhibitor, verapamil and the MRP inhibitor, MK571 were purchased from Cal-biochem (San Diego, CA). The P-gp specific inhibitor, PSC-833 was purchased from Xenotech (Lenexa, KS). Radio-labeled [^3^H]-saquinavir (^3^H-SQV) was obtained from Moravek Biochemicals (Brea, CA) and unlabeled saquinavir mesylate (Invirase™) was obtained from Tulane University hospital pharmacy (New Orleans, LA). The Trizol™ reagent for RNA isolation was obtained from Invitrogen (Carlsbad, CA) and diethylpyrocarbonate (DEPC) treated water was purchased from Ambion (Austin, TX). Reagents for reverse transcription (RT), e.g. M-MLV reverse transcriptase, oligo deoxythymidine (oligo-dT) primers and RNAase inhibitor, were purchased from Promega (Madison, WI). All other Polymerase Chain Reaction (PCR) reagents, such as buffers, deoxy-nucleotide triphosphates (dNTPs) and the Taq DNA-polymerase were obtained from Sigma Aldrich (St. Louis, MO). The PCR primers were synthesized from Midland Certified Reagent Company (Midland, TX). Cell lysis buffer for protein isolation was obtained from Cell Signaling (Danvers, MA) and the BCA protein assay kit was purchased from Pierce (Rockford, IL). Mouse monoclonal anti-MDR1 (P-gp) antibody (Clone #C-219) was purchased from Abcam Inc. (Cambridge, MA) and the goat anti-mouse secondary antibody (A9044) was obtained from Sigma. The PKC pathway stimulator phorbol-12-myristate-13-acetate (PMA) was also obtained from Sigma. For immunocytochemistry studies, Alexa fluor-488 conjugated goat anti-mouse antibody and Prolong antifade reagent, were both purchased from Invitrogen (Grand Island, NY). The control and MDR1 specific short interfering RNAs (siRNA) and transfection reagents were obtained from Dharmacon (Lafayette, Co). The HIV-1 p24 Antigen Capture ELISA kit was purchased from ZeptoMetrix Corporation (Buffalo, USA).

### Cell Culturing

The primary brain endothelial cells (HBMVEC), cell culture media, cell attachment factor and cell passage reagents were all purchased from Cell Systems (CS-C) (Kirkland, WA). The HBMVEC were maintained in CS-C Complete medium, according to the supplier’s recommendations, in a 37°C incubator with 5% CO_2_. For experiments, the HBMVEC were plated in 75 cm^2^ flasks coated with CS-C Attachment Factor and cultured in CS-C media containing 10% fetal bovine serum (FBS), CS-C Growth Factor and 2% gentamycin sulfate. Experiments were conducted within cell passages 4–6. At these passages, cells displayed cobblestone appearance which is morphologically correct for ECs. All treatments were performed after cells were 60–70% confluent. The HuT78 T-lymphocytic cell line was obtained from American Type Culture Collection (ATCC, Manassas, VA) and the HTLV-IIIB (HIV-1-infected H-9 T-cells) (Cat. No. -398) was obtained from NIH AIDS Reagent Program (Germantown, MD, USA). Both lymphocytic cell lines were maintained in RPMI-1640 medium containing 10% FBS and 2% penicillin/streptomycin, and up to passages 12–15 were used for all experiments. To confirm the consistent productive infection in these T-cells, supernatants from infected cells were regularly monitored for HIV-1 p24 levels by ELISA.

### Virus Preparation, Infection and Heat Inactivation

Cell-free viral stock was obtained by harvesting the supernatants of HTLV-IIIB cells, centrifugation at 10,000 rpm for 10 min and filtration through a 0.2 µ filters. The concentration of viral particles was determined by p24 ELISA and all HIV-1 exposure studies were performed with viral inoculums of 0.2 or 1.0 multiplicity of infection (MOI) where a stock concentration of ∼100 pg/ml p24 was designated as 1.0 MOI. Heat inactivation of HIV-1 was performed by heating the cell free viral stocks at 65°C for 45 min, and the absence of infectious virus after heat inactivation was corroborated by incubation with uninfected HuT78 cells followed by p24 ELISA at 3 and 5 days post infection [13].

### Preparation of the Blood Brain Barrier Model

The HBMVEC were seeded on 0.4 µm pore size PTFE (Polytetrafluoroethylene) membrane tissue culture inserts (Corning, NY) at the initial concentration of 10^4^ cells/well, as previously described [12]. For infection experiment, HBMVEC were allowed to grow up to 70% confluency and then the viral inoculum was added to the upper chamber of the trans-well inserts. After 24 hrs of incubation, HBMVEC were washed thoroughly with sterile PBS and replenished with fresh media. For drug exposures, i.e. saquinavir, verapamil, MK-571 or PSC-833, all drugs were added to the upper chamber of the trans-well inserts, at the specified times and for the specified durations and cells were harvested at different time points for mRNA and protein isolation as well as for fluorescence microscopy. At the beginning of each experiment, and at different times post-exposure, quantitation of HIV-1 viral particles in the culture supernantants was analyzed via p24 ELISA.

### Calcein-AM Efflux Assays

Calcein-AM, a lipid-soluble dye and a known substrate for both MDR1 and MRP transporters, was used to determine MDR1 and MRP-mediated efflux functions (Molecular Probes; Eugene, OR) [14], [15]. Upon entering cells, endogenous esterase cleaves calcein-AM to form the hydrophobic fluorescent calcein. ABC-transporters cause rapid efflux of the dye prior to esterification and intracellular calcein-AM florescence can be increased in presence of ABC-transporter inhibitors, e.g. verapamil or MK-571. Hence, intracellular retention of this fluorochrome is inversely proportional to the extent of cellular efflux and the effect of inhibitors indicates the contribution of different transporters. For this assay, HBMVEC were cultured in 24-well opaque black plates (Krystal, Labnet, NJ) and treatments such as virus and drug exposures were carried out as in previous section. To monitor effects on drug-efflux function, cells were incubated for 15 min with verapamil or MK-571 (50 µM) at 37°C. Calcein-AM (0.25 µM) was then added to cells and incubated at 37°C for 15 min in the dark, followed by three washes with ice-cold phosphate buffered saline (PBS). Intracellular calcein-AM fluorescence (retention) was determined using a FLx800 fluorimeter (Bio-Tek instruments) with absorption and emission wavelengths set at 485±20 nm and 528±20 nm, respectively. Subsequently, cell lysis was done by adding 1X lysis buffer (30 µl) to each well for 10 min and the extracted proteins were quantified using the BCA protein assay kit (Thermo Scientific, IL, USA). Fluorescence measurements obtained from each well were normalized to the protein concentration in the respective samples and are represented as fluorescence/mg of protein. Differences in calcein-AM retention with and without verapamil or MK-571 indicated the efflux function in control and virus and/or drug exposed HBMVEC.

### RNA Isolation and Semi-quantitative RT-PCR Assays

Total RNA was isolated from cells using the Trizol™ reagent (Invitrogen; Carlsbad, CA) according to the manufacturer’s instructions. The RNA concentrations were quantified using a spectrophotometer to determine the absorbance at 260 nm and purity was verified by the 260/280 nm absorbance ratio. RNA aliquots were frozen at −80°C for later use in reverse transcriptase polymerase chain reaction (RT-PCR) assays. Briefly, total RNA (2 µg) was first subjected to first-strand cDNA synthesis using a high capacity cDNA reverse transcription kit (Applied Biosystem, USA) using the murine leukemia virus (MuLV) RT enzyme (0.5 U), Oligo-dT (2.4 µg/ml), and dNTPs (100 mM) and incubation at 42°C for 60 min. For each PCR amplification reaction, 3 µl of this RT-product was used which were carried out by using a thermal cycler from Perkin Elmer (Boston, MA; Model 9600) in the presence of *Taq* DNA-polymerase (0.5 U) in RED-Taq PCR buffer, containing KCl (500 mM), MgCl_2_ (11 mM), dNTPs (200 µM) and the gene-specific primers. The primer sequences and amplification conditions for MDR1, MRP-1 and MRP-2 were according to Hua *et al* (2005) [16] and for GAPDH according to Konig *et al* (2005) [17]. The PCR products were electrophoresed on a 2.0% agarose gel containing ethidium bromide (10 µg/ml) along with a molecular weight marker DNA ladder (100–3000 bp) (SM-0243; Fermentas, USA). Band intensities for PCR products were determined by densitometry by using a GS-700 imaging densitometer (Bio-Rad). The intensity of amplified ABC-transporter mRNAs were normalized to the values obtained with GAPDH mRNA in each sample.

### Quantitative RT-PCR Assays

Qantitative PCR assays using SyBR Green dye was performed with a Perkin-Elmer 7700 thermal cycler. Briefly, total RNA was extracted and quantified as described above. For quantification of each ABC transporter, 10 µl of reaction mixture containing RNA (2 µg) was added to each well of a 96-well optical reaction plate (Applied Biosystems, USA). Primer sequences and amplification protocols for quantitative analysis of MDR1 and GAPDH (internal control) mRNA levels were as previously published [18], which was as follows: 2 min at 50°C, 10 min at 95°C, followed by 45 cycles of 15 sec at 95°C and 1 min at 60°C. Post run calculations were done according to the manufacturer’s software. The obtained Ct values for each ABC transporters (MDR1, MRP-1, MRP-2 & MRP-3) in both control and HIV-1 and/or SQV exposed HBMVEC, were normalized with GAPDH mRNA levels. The Ct values were further normalized to each of the transporter genes in uninfected control cells and graphical presentation of delta-delta (ΔΔ) Ct values are displayed as the normalized fold changes.

### Saquinavir Accumulation Assays

Tritium (^3^H-) labeled SQV was used to measure the intracellular accumulations of this HPI in control and HIV-1 exposed HBMVEC. A non-specific inhibitor, verapamil and a MDR1 specific inhibitor PSC-833, were used to determine the efflux rates of ^3^H-SQV from cells. Briefly, cells were grown to 70% confluency in 24-well culture plates and were exposed to HIV-1 and/or SQV for the indicated time points. To measure efflux rates, cells were first incubated with verapamil or PSC-833 (50 µM) for 15 min, followed by a 2 hr exposure to ^3^H-SQV (1.7 pM; specific activity: 1.0 Ci/mMol). To determine the intracellular retentions, in the presence or absence of MDR-specific inhibitors, cells were washed twice with ice cold PBS and cell extracts were obtained by lysing cells with 1.0 ml of NH_4_OH (1.0 M) for 5 min. Approximately 500 µl of cell extracts were transferred to scintillation vials containing 10 ml of Ecolite scintillation cocktail and 200 µl of extracts were used to measure total protein levels. The counts per minute (CPM) values in cell extracts were determined with a Tri-Carb 2800TR Liquid Scintillation counter (Perkin Elmer, USA) and the data were normalized to the protein contents in respective samples, and represented as CPM/µg of protein. Differences in ^3^H-SQV retention with and without verapamil or PSC-833 demonstrated drug-efflux rates in control, and in SQV and/or HIV-1 exposed HBMVEC.

### Western Blot Analysis

The HBMVEC were grown in 10 cm cell culture dishes coated with collagen, exposed to HIV-1 and/or SQV for the indicated concentrations and times, and washed twice with PBS before harvesting proteins using ice-cold lysis buffer. Equal amounts of proteins (60 µg) were loaded in 7.5% polyacrylamide (SDS-PAGE) gels and subsequently transferred to a nitrocellulose membrane. For hybridization, membranes were first incubated with 5% milk for 1 hr, washed, and incubated overnight at 4°C with a mouse monoclonal anti-human antibody against MDR1 (C-219, Abcam, CA) at a 1∶200 dilution. After incubation, membranes were thoroughly washed and incubated with Horseradish peroxidase (HRP) conjugated goat anti-mouse secondary antibody (1∶5000 dilution) for 1 hr. A rabbit anti β-actin antibody (Sigma, USA) was used as internal control (1∶8000 dilutions). HRP was detected by using a chemiluminescent detection system (LumiGLO, Gaithersburg, MD). Densitometric analysis of the respective protein bands was performed using a imaging software (Bio-Rad).

### Immunofluorescent Microscopy

Cells were grown on collagen-I coated four chambered slides (BD Biosciences) to approximately 60–70% confluency and then processed as published previously [19]. Following their exposure to HIV-1 and/or SQV for 24 hrs, cells were incubated with MDR-1 specific primary antibody and then with FITC-conjugated secondary antibody. Cell nuclei were visualized with 4′,6-diamidino-2-phenylindole hydrochloride (DAPI) (Molecular Probes, Eugene, OR) and a wheat gram agglutinin (WGA) conjugated to AlexaFluor-555 (Molecular Probes, USA) was used as a cell surface marker. Primary antibodies were used at a dilution of 1∶20 and secondary antibodies at 1∶200 dilution. Images were acquired with a Zeiss Axioplan II microscope (Carl Zeiss, Thornwood, NY). A series of horizontal optical sections (0.3 µm each) were collected and subsequently deconvolved using Slidebook 4.1 (Intelligent Imaging Innovations, Denver, CO) or Volocity (Improvision, Lexington MA) softwares. Images represent a merging of 10–15 sections (3–4.5 µm). Post-collection processing of the images was performed using Adobe PhotoShop.

### siRNA Transfection

The HBMVEC were transfected with either a non-specific (random) or a MDR1-specific siRNA cocktail (smart pool) and siRNA transfection reagents (Dharmacon, USA) and according to the manufacturer’s instructions. Briey, cells were seeded in six-well plates at a density of 1×10^5^ cells per well until 70% conuent. Cells were then treated with HIV-1 (0.2 MOI) and SQV (0.3 µM) for 24 hrs. Following the incubation, cells were washed with complete medium (antibiotic-free). Required concentrations of siRNA and transfection reagent were prepared separately, mixed together for 15 min and added to the cells containing 2 ml media for 24 hrs and incubation at 37°C with 5% CO_2_. Four types of established MDR1-specific siRNAs (30 nM each) were used as the smart pool. Random siRNA oligos and GAPDH siRNAs were used as negative and positive controls, respectively. After incubation, one set of cells were harvested for RNA extraction, cDNA was synthesized with respective RNA samples and RT-PCR was carried out, as described previously. To perform quantitative PCR amplifications, serial dilutions of each cDNA samples were used. GAPDH was used as an internal control. The Ct values were calculated for each sample and graphical representations were done with respect to percentage inhibition of MDR1 gene with 30 nM siRNA compared to control (untreated) cells. Parallel wells containing siRNA transfected HBMVEC were also analyzed for changes in drug-efflux functions using calcein-AM, as previously described.

### Statistical Analysis

All statistical analyses were carried out using the INSTAT-2 software (Graph Pad, San Diego, CA). Each treatment condition consisted of three to four replicates and each experiment was performed at least three to five times. Data obtained were used to calculate the standard error of means (± SEM). Significant changes from control values were determined by using a two-tailed student’s t-test and comparison between three or more groups was carried out by one-way analysis of variance (ANOVA).

## Results

### MDR1 Gene Expression Significantly Increased in HBMVEC Following Exposure to HIV-1

**Figure 1:**
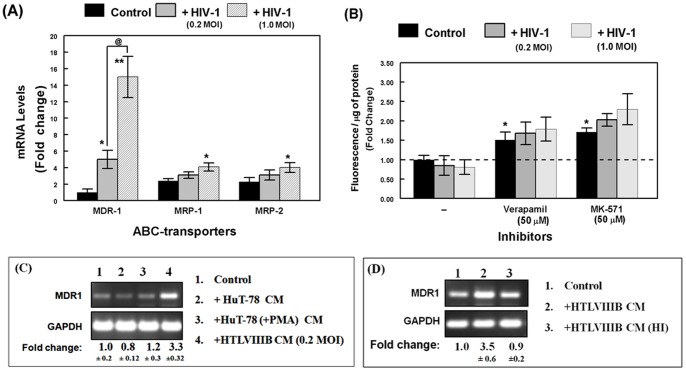
Effect of HIV-1 exposure on MDR1 gene expression and efflux function in HBMVEC. (**A**) MDR-1, MRP-1 and MRP-2 gene expression following exposure to HIV-1 (0.2 and 1.0 MOI) within 6 hrs post exposure was monitored by qRT-PCR. Data obtained in each sample was normalized to GAPDH mRNA levels (internal control). Fold change in ABC-transporter gene expression with respect to that observed with MDR1 in untreated cells (1.0) was calculated under each treatment condition and for each transporter. Data represent three independent experiments (n = 3) where standard deviation (error bars) were calculated from triplicate samples and significant differences from controls are shown (*, p<0.05 and ** p<0.01). (**B**) MDR1 efflux function following exposure to HIV-1 (0.2 and 1.0 MOI) for 24 hrs. Verapamil and MK-571 were used as MDR1 and MRPs blocker, respectively. Intracellular fluorescence was normalized to total protein content in cell lysates and change in calcein-AM retention is shown in the bar graphs (*, p<0.05). (**C**) Comparative effects of conditioned medium (CM) from HIV-1-infected or PMA-stimulated HuT-78 T-cells on MDR1 gene expression. The RT-PCR products from HBMVEC control (1); Hut-78 CM (2); CM from HuT-78 cells treated with PMA (3); or CM from virus producing HTLV-IIIB cells (4), are shown. (**D**) Effect of live HIV-1 (HTLV-IIIB CM) and heat inactivated HIV-1 (HTLV-IIIB CM-HI) on MDR1 gene expression. The respective fold change in MDR1 gene expression was calculated with respect to GAPDH (internal control). A representative data from three independent experiments, is shown.

To elucidate the transporters which are mainly involved in drug efflux in the HBMVEC, cells were exposed to HIV-1 (0.2 or 1.0 MOI) for 6 hrs. The real-time RT-PCR showed a 12–18 fold increase in MDR1 gene expression within 6 hrs post exposure to cell free virus. However, the MRP1 and MRP2 expressions were only increased by 1.5 to 2 folds (Fig. 1A). Furthermore, the drug-efflux function in HIV-1 exposed HBMVEC was determined after 24 hrs post virus exposure by calcein-AM retention assay (Fig. 1B). Exposure to either the dual (P-gp and MRP) inhibitor, verapamil or the MRP-specific inhibitor, MK571 (50 µM each) increased intracellular fluorescence in control cells, suggesting a constitutive low level of drug-efflux function in HBMVEC. Interestingly, even though virus exposure had resulted in significant increases in MDR1 and MRP gene expressions, the drug efflux function via these transporters were not significantly augmented, as apparent from minimal changes in calcein-AM retention as compared to control cells (Fig. 1B). Therefore, for the rest of our studies, further characterization of HIV-1 induced changes in drug-efflux function was focused on MDR1 only.

In order to find out whether the virus alone, virus-induced inflammatory cytokines, or host cellular factors, are responsible for the augmenting effects on MDR1 gene expression, the HBMVEC were exposed to culture media from unstimulated or PMA-stimulated Hut78 cells, or with cell free virus which was heat inactivated. MDR1 gene expression was significantly higher (3.3 fold) only when HBMVEC were exposed to live virus, but not with either the heat-inactivated viral supernatant or the PMA-stimulated culture supernatants (Fig. 1, C & D). This clearly indicated the direct role of infectious virus in increasing MDR1 gene expression in HBMVEC.

### Both MDR1 Protein Level and MDR-1 Mediated Drug-efflux were Increased in HBMVEC Following Co-exposure to HIV-1 and Saquinavir

**Figure 2:**
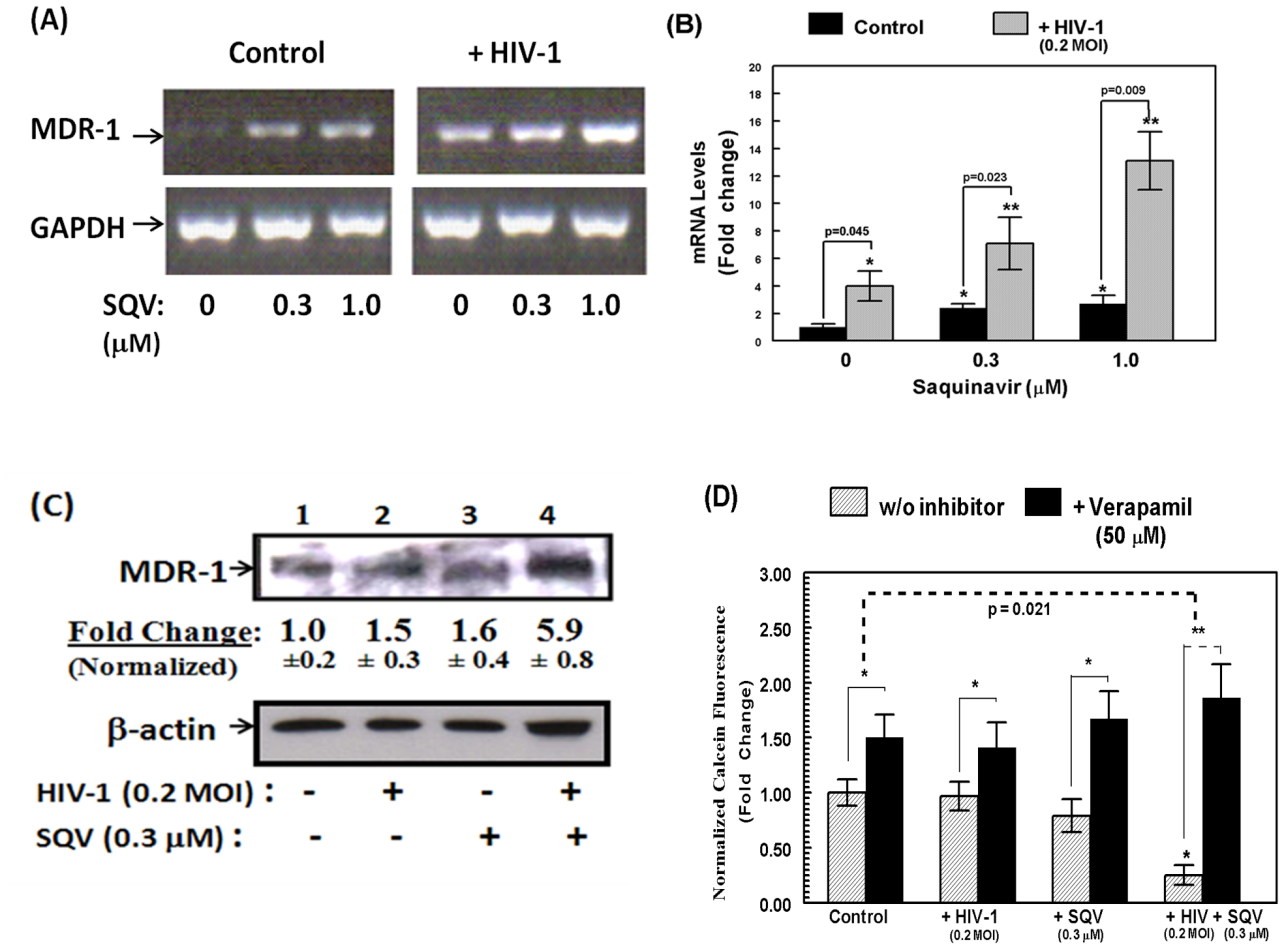
Effect of HIV-1 and SQV coexposure on MDR1 expression and function in HBMVEC. (**A**) MDR1 gene expression in presence of HIV-1 (0.2 MOI) and increasing concentration of SQV (0.3 and 1.0 µM). (**B**) Densitometric analysis of mRNA expression from Fig. 2A. (**C**) A representative of western blot analysis data of MDR1 protein expression in untreated (1), HIV-1 (2), SQV (3) and HIV-1+ SQV (4) exposed cells. Beta (β)-actin was used as internal control. The fold change analysis was done from three sets of independent western immunodetection studies. MDR1 protein expressions are normalized with β-actin expression in respective samples. (**D**) MDR1 mediated calcein-AM efflux in presence of HIV-1 (0.2 MOI) and/or SQV (0.3 µM). Grey bars indicate calcein retenition without inhibitor and black bars indicate calcein retention in presence of 50 µM verapamil. Data shown are mean of three independent experiments (*, p<0.05 and **, p<0.01).

In order to observe the combined effects of s infectious HIV-1 and the anti-HIV drug, saquinavir (SQV) on MDR1 expression and efflux function, cells were exposed to HIV-1 (0.2 MOI) alone and in the presence of SQV (0.3 or 1.0 µM). The fold change values on MDR1 gene expression, normalized with GAPDH expression, showed that exposure to even low MOI of HIV-1 increases MDR1 mRNA levels, whereas the combined effect of HIV-1 and SQV (1 µM) showed much higher induction of the MDR1 gene (Fig. 2A & 2B). The densitometry analysis also indicated that with increasing concentration of SQV, MDR1 gene expression successively increased along with co-exposure of HIV-1 (Fig. 2B). We investigated whether the MDR1 gene activation was translated to increased protein levels. Western blot analysis showed that, compared to control (untreated) cells, there was an increase in protein expression following exposure to SQV (0.3 µM) and HIV-1 (0.2 MOI), both separately and in combination (Fig. 2C). To obtain fold-change ratios, protein expression from untreated samples were empirically designated as 1.0. Exposure to SQV and virus showed almost 1.6-fold increase in protein levels, but in combination showed approximately 5.9-fold increase in MDR1 protein level (Fig. 2C). The above data suggested that co-incubation with the drug SQV and/or virus has synergistic effects on the MDR1 gene as well as protein expression levels. Therefore, we investigated whether augmented drug efflux function was seen in HBMVEC exposed to HIV-1 (0.2 MOI) and SQV (0.3 µM). Calcein-AM efflux assays were carried out in presence or absence of verapamil (50 µM). Efflux function was fully suppressed when cells were pre-incubated (15 min) with verapamil before the addition of calcein-AM. The differences in calcein-AM fluorescence indicated the drug-efflux function under each treatment condition. The change in calcein retention was almost 10-fold (from 0.17 to 1.5) in cells co-exposed to HIV-1 and SQV, but was less than 2-fold in both untreated cells and in cells exposed to either the virus or SQV alone (Fig. 2D). This data clearly established that coexposure to both drug and virus contribute to the increased efflux activity of MDR1 in HBMVEC.

### Immunofluorescence Microscopy Revealed Up-regulation of Membrane MDR1 Levels Following Co-exposure to HIV-1 and SQV in HBMVEC

**Figure 3:**
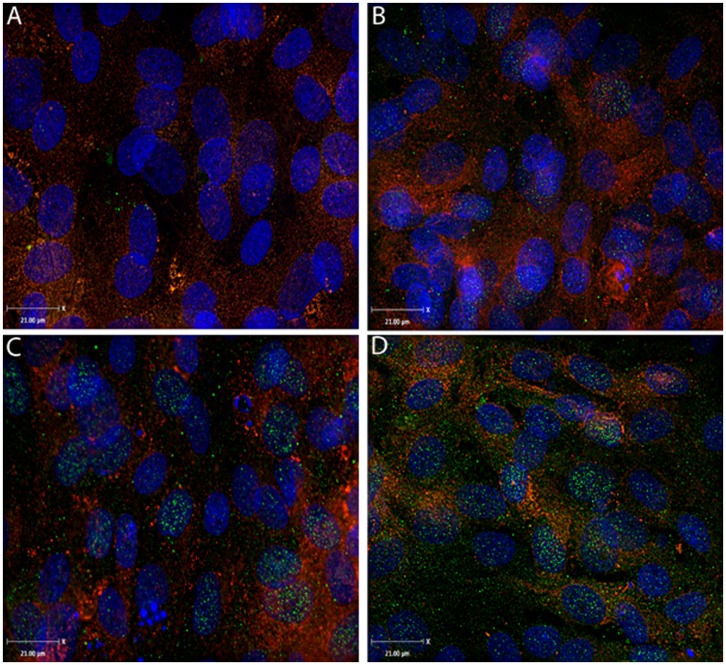
Localization of MDR-1 (P-gp) protein in HIV-1 and SQV coexposed HBMVEC. A representative immunofluorescence microscopy data of MDR-1 protein in HBMVEC following exposure to HIV-1 or SQV for 24 hrs is shown. Green punctate MDR-1 staining appeared in (**A**) untreated cells, (**B**) cells exposed to SQV (1 µM), (**C**) cells exposed to HIV-1 (0.2 MOI) only, and (**D**) combined exposure of SQV and HIV-1. DAPI (blue) staining of the nucleus and WGA (red) staining of cell plasma membrane are also shown. Image Magnification: 630X, scale bar: 21 µm.

To further corroborate our findings that HIV-1 and SQV co-exposure results in increased MDR1 protein expression, cells were treated with either HIV-1 (0.2 MOI) or SQV (1.0 µM) separately or in combination for 48 hrs. Following which cells were stained with antibodies to visualize the expression and sub-cellular localization of MDR1 using a fluorescent microscope with confocal imaging capability (Fig. 3). Cell nuclei were visualized by DAPI staining (blue) and membrane staining was carried out using WGA (red). A punctate staining pattern of MDR1 (in green) was observed regardless of treatment (Fig. 3). To confirm membrane localization of the protein, WGA was used to stain the cell surface. Analysis of the Z-stack in overlaid pictures indicated similar localization of WGA and MDR1, strongly suggesting a membrane-associated pattern of MDR1 expression. As compared to untreated cells (Fig. 3A), a more intense staining for MDR1 was observed when cells were exposed to either HIV-1 (Fig. 3B) or SQV (Fig. 3C) which was further increased by co-exposure of HIV-1 and SQV (Fig. 3D). These observations corroborated the gene and protein expression data, and indicated that concomitant exposure to HIV-1 and SQV increases MDR1 expression at both the transcriptional and translational levels, and facilitates their membrane localization which is required for their drug-efflux functioning.

### Co-exposure to HIV-1 and SQV Increased MDR1-mediated Efflux of Radiolabeled Saquinavir (^3^H-SQV) in HBMVEC

**Figure 4:**
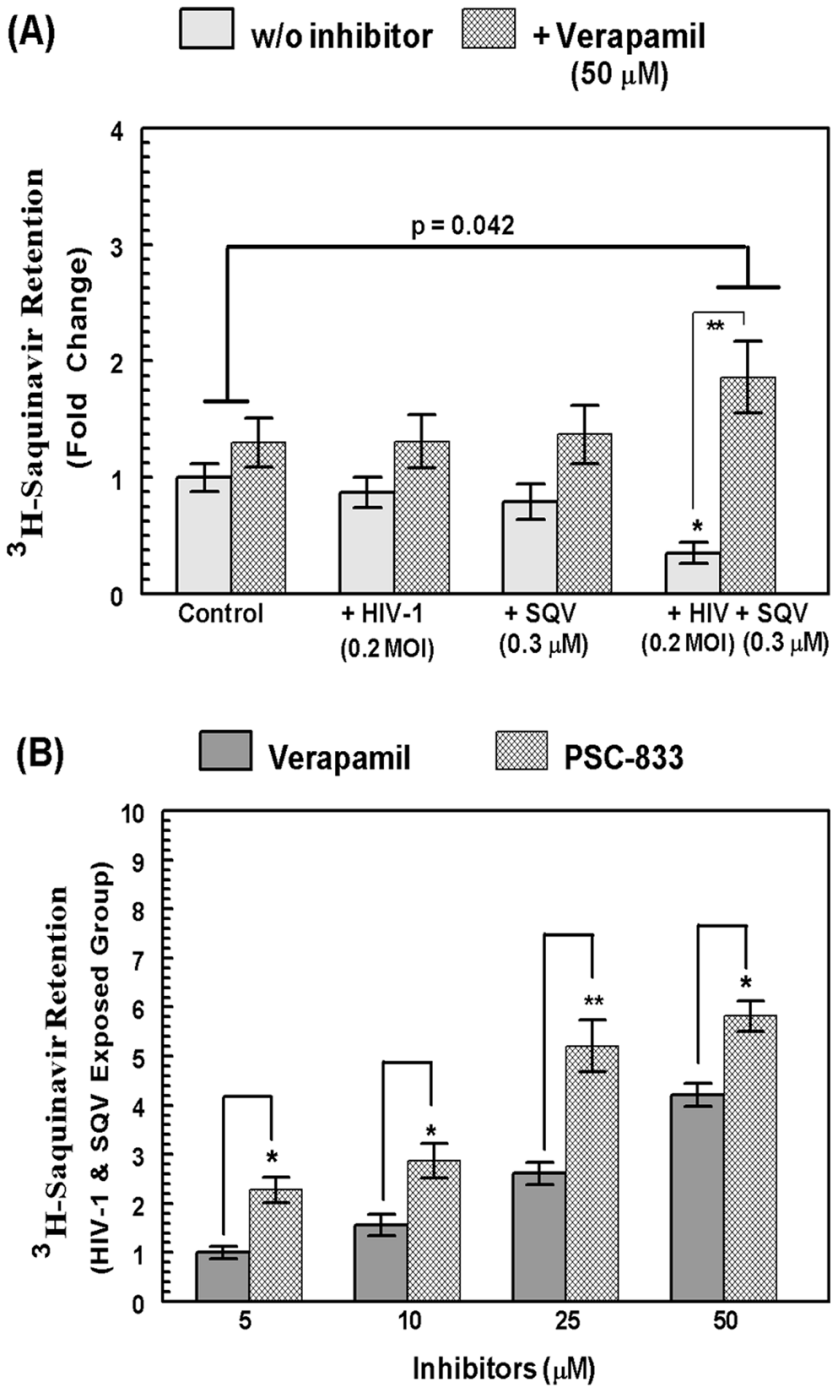
A comparative analysis of ^3^H-SQV efflux in HBMVECs exposed to HIV-1 and/or SQV in presence of verapamil or PSC-833. (**A**) Effect of verapamil (50 µM) on ^3^H-SQV retention in HBMVEC exposed to HIV-1 and/or SQV for 24 hrs. Under each treatment condition, intracellular ^3^H-SQV was measured in presence of verapamil and compared with control (without inhibitor). The radioactivity in cell lysates were normalized to total cell protein content (CPM/ug of protein) and data from three independent experiments are presented as fold changes. (**B**) Effect of increasing concentrations (5–50 µM) of verapamil or PSC-833 on ^3^H-SQV retention in HBMVEC exposed to HIV-1 and SQV for 24 hrs. Data are representative of three separate independent experiments carried out in triplicates (*, p<0.05 and **, p<0.01).

In order to determine whether the increased MDR1 expression in cells coexposed to HIV-1 and SQV can indeed enhance the functional activity of MDR1 in extruding HPIs from HBMVEC, efflux function was measured by SQV retention, since it is a known substrate of MDR1 [20]. Tritium labeled SQV (^3^H-SQV) was used to monitor the drug efflux rates under untreated and HIV-1-exposed conditions, in the presence or absence of verapamil (50 µM). Intracellular radioactivity was measured as counts per minute (CPM) after incubation under different conditions and is considered as the level of ^3^H-SQV retention. Compared to control conditions, there was a significant decrease in SQV retention in the ^3^H-SQV and HIV-1 coexposed cells, and significant increase (p<0.042) was observed when cells were pretreated with verapamil (Fig. 4A). The combined exposure to HIV-1 and SQV showed a 3.5-fold difference in ^3^H-SQV retention with and without verapamil. The graphs show intracellular ^3^H-SQV contents, normalized to the total protein contents in respectively treatment wells. These drug-efflux assays clearly exhibited the increased activity of MDR1 and SQV efflux under HIV-1 and HPI exposed microenvironments. Since verapamil can be a dual inhibitor of both MDR1 and MRPS, in order to further verify the crucial role of MDR-1 transporter, ^3^H-SQV retention was carried out in the presence of increasing concentrations (5–50 µM) of verapamil or the MDR-specific inhibitor PSC-833 (Fig. 4B). The specific increase in MDR1-mediated efflux following HIV-1 and SQV coexposure was clearly evident from findings using PSC-833. The intracellular ^3^H-SQV retention was considerably higher with PSC-833 compared to the verapamil pretreated cells, at all tested conditions. This clearly indicated a direct role for the MDR1 transporters in increased drug efflux from HBMVEC.

### siRNA-mediated MDR1 Inhibition Suppresses Drug-efflux Function in HBMVEC Cells Co-exposed to HIV-1 and SQV

**Figure 5:**
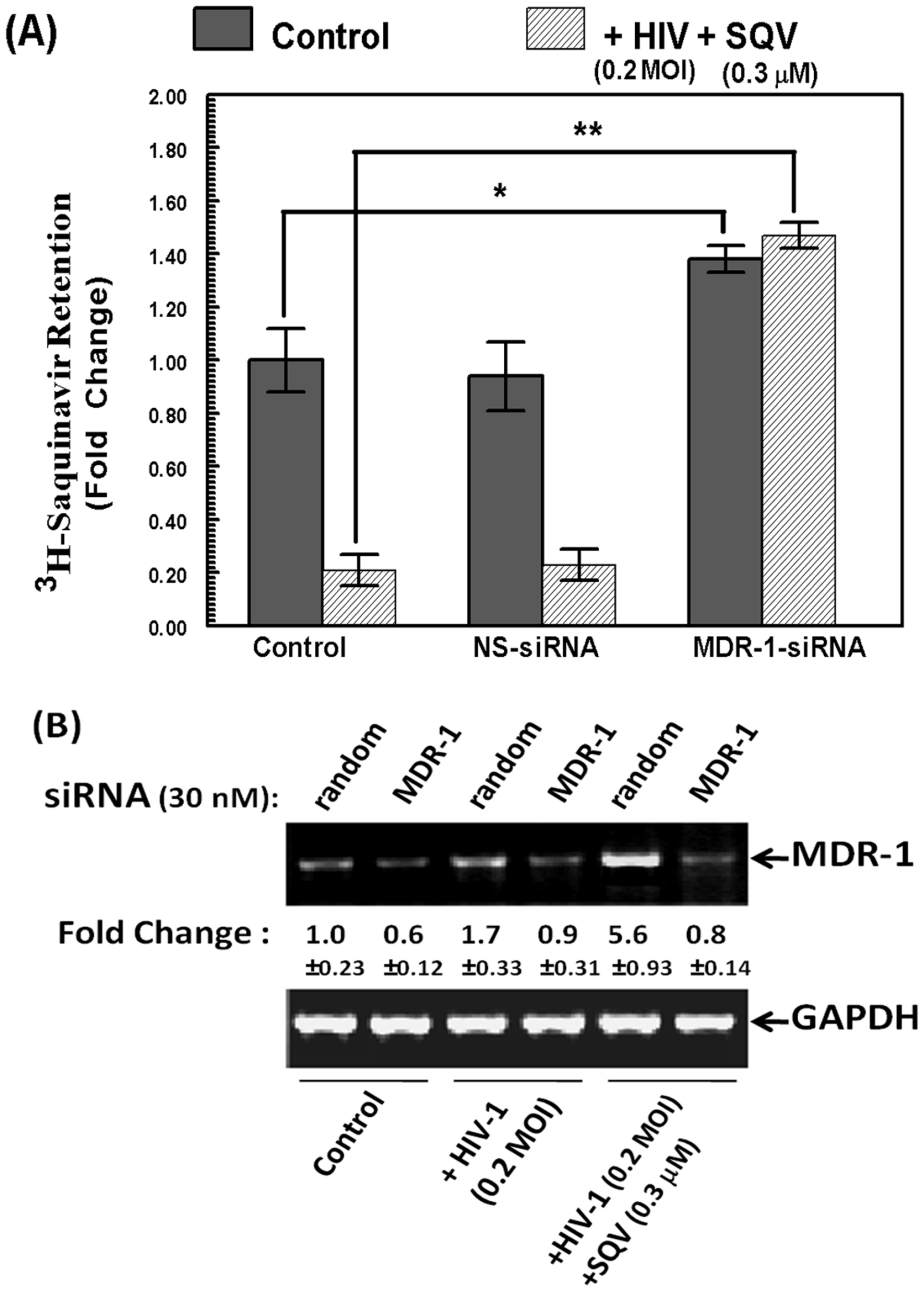
Effect of MDR1 specific siRNA on HIV-1 and SQV induced drug efflux in HBMVEC. (**A**) Fold change in ^3^H-SQV retention (CPM/µg of protein) in HBMVEC are shown in control (untreated) and HIV-1 (0.2 MOI) and SQV (0.3 µM) exposed cells. Data from non-transfected, and in cells transfected with either a random (NS) or an MDR1 specific siRNA, are shown. Data are representative of three independent experiments carried out in triplicates (*, p<0.05 and **, p<0.001). (**B**) Relative inhibition of MDR1 gene expression in MDR1 siRNA transfected and control (random) siRNA transfected cells are shown by semi-quantitative RT-PCR. Change in MDR1 gene expression following treatment with HIV-1 (0.2 MOI) and HIV-1+SQV, in control and siRNA transfected cells, are shown. Changes in band intensities of PCR products for MDR1 mRNA were normalized to the GAPDH mRNA levels, in respective samples, and fold changes obtained compared to control (1.0) are shown.

To further corroborate the role of MDR1 only, the HBVECs were transfected with either a non-specific (NS) siRNA or a smart-pool of siRNAs specific for the human MDR1 (30 nM each). After 24 hrs, cells were then exposed to the combined regimen of HIV-1 (0.2 MOI) and SQV (0.3 µM) for another 48 hrs. In one aliquot of cells, we measured intracellular retention of ^3^H-SQV (Fig. 5A) and in a parallel batch of cells we measured the extent of siRNA mediated MDR1 inhibition by RT-PCR (Fig. 5B). Data obtained in control (untreated) and HIV-1 plus SQV exposed HBMVEC clearly depicted approximately 5-fold decrease in intracellular ^3^H-SQV levels in cells transfected with the NS-siRNA, which was similar to cells not transfected with any siRNAs (Fig. 5A). However, the direct involvement of MDR1 in drug efflux was clearly apparent by an almost total suppression of ^3^H-SQV efflux in cells transfected with the MDR1 siRNA. Indeed, the MDR1 siRNAs were able to increase the intracellular ^3^H-SQV levels even in untreated cells (p<0.05), indicating that HBMVEC possess a MDR1 mediated constitutive drug-efflux mechanism which is significantly up-regulated when exposed to HIV-1 and subsequently to the anti-HIV agent, SQV. The constitutive and inducible expression of MDR1 is also clearly evident from the semi-quantitative RT-PCR analysis (Fig. 5B). A detectable expression was seen in untreated cells, HIV-1 exposure resulted in a 2-fold increase, and co-exposures to HIV-1 and SQV caused a higher than 5-fold increase in MDR1 mRNA levels. The potency of siRNA mediated inhibition of MDR1 mRNA levels was evident by the RT-PCR investigations in cells transfected with 30 nM of random (NS) siRNA or MDR1 siRNA. There was a significant inhibition of MDR1 gene in both control (60%), HIV-1 (56%) and HIV-1 plus SQV (86%) exposed cells. This data clearly indicated that MDR1 is specifically responsible for the increased drug-efflux function in HIV-1 and SQV coexposed HBMVEC.

## Discussion

The HIV-1 infected cells enter the brain very early following infection via traversing the BBB endothelial cells and these reservoirs persist within the CNS [21]. In this regard, finding efficient ways to deliver HAART drugs to these brain reservoirs of HIV-1 would be very crucial, to both suppress disease progression and selection for drug resistance. Indeed, MDR1 is an important drug-efflux mechanism expressed at the BBB, and is well known to limit HAART efficacy in the CNS. Other transporters which co-exist with MDR1 on the apical (blood) side, e.g. MRPs, might also play a role in reducing drug efficacy. However, in our study the major contribution of HIV-1 and SQV coexposure was found to be via the MDR1 pump. The initial gene expression study which indicated the inductive effect of HIV-1 on transporter expressions, was evident within six hours of exposing cells to virus-containing media. There was an immediate induction of MDR1, and both MRP-1 and MRP-3 genes. More importantly, MDR1 gene expression was found to be significantly greater than the other two transporters investigated in this study. The inductive role of HIV-1 was further investigated to determine whether other contributing factors in the virus infected cellular supernatants can increase MDR1 gene expression. However, supernatants from PMA-stimulated HuT-78 lymphocytes, which produce inflammatory cytokines, showed negligible effects on MDR1 expression. Surprisingly, inflammatory factors could not induce MDR1 gene expression as compared to that observed with supernatants containing live virus, which was clearly evident when virus was heat-inactivated in the supernantants (Fig. 1C). The MDR1 gene expression with increasing concentration of virus (from 0.2 to 1.0 MOI) was further investigated towards MDR1-mediated efflux function, which did not change significantly in cells exposed to virus only. This definitely confirmed the role of live virus in MDR1 gene expression, and increased efflux activity of MDR1 is not achieved by virus exposure only. Although, it has been reported that MDR1 transporter function may depend on host cellular factors such as inflammatory cytokines [22], [23] and the HIV-1 Tat protein has also been shown to increase MDR1 expression in a murine model [24], [25], our findings revealed new information about the direct role of HIV-1 in MDR1 induction on HBMVEC, especially under concomitant HPI exposure. According to our knowledge, this is the first time a synergistic role of virus and anti-HIV drugs has been documented on HBMVEC under *in vitro* conditions. The current study also showed that inflammatory factors from activated lymphocytes had negligible effects on MDR1 induction which further indicates that the presence of infectious HIV-1 is mainly responsible for the increase in MDR1 gene expression and concomitant exposure to the MDR1 substrates, such as the anti-HIV drugs, significantly increases MDR1 activity.

Indeed, MDR1 substrates include most of the currently available HPIs, and our previous research showed that SQV induces MDR1 function [8]. The RT-PCR data showed a significant increase of MDR1 following exposure to HIV-1 and/or SQV. Moreover, with increasing concentration of SQV, we observed that MDR1 expression was also increased. This data indicated that MDR1 may definitely contribute to HAART drug resistance mechanisms within the CNS reservoirs of HIV-1. The MDR1 protein expression in HBMVEC was also changed under different treatment conditions (Fig. 2C). Primarily, the synergistic effect of drug and virus coexposure significantly increased the MDR1 protein level, membrane localization, and efflux function. Thus, our findings strongly suggest that the synergistic effect of HIV-1 and SQV has induced certain cellular pathways that in turn up-regulate MDR1 expression and efflux function, although both of these factors were also seen to independently induce the MDR1 protein level. Previous studies have shown the involvement of different host cellular factors such as cytokines and chemokines (TNF-α, IL-6, etc.) in MDR1 regulation in different human cell types [4], [10], [24], [26], [27]. In HIV infected patients, inflammation alters MDR1 expression in the brain and numerous chemokines are believed to be the inflammatory mediators [28]. It was observed that increase in chemokines in humans may increase the MDR1 expression in BBB endothelial cells through different signaling pathways, mainly the protein kinase-C (PKC) pathway [6], [29]. However, recent studies on rat brain capillaries indicated that there is a network of signaling mechanisms that modulate MDR1 activity involving TNF-α and IL-6 [29], [30]. On the other hand, HIV-1 Tat protein was also found to play an important role in overexpressing MDR1 in rodent BBB by activating NF-κB [31]. The Tat-induced over expression of MDR1 in brain microvessels clearly indicated the role of HIV-1 in MDR1 regulation. Likewise, in the present study, the primary induction in MDR1 following exposure to HIV-1 emphasizes that the virus mainly has an effect on MDR1. So far, most of the previous HIV-1-related MDR1 induction studies were conducted in mice or rat cells that may not be relevant in human cells, because most host immune responses vary among species. The present study demonstrates that viral induction of MDR1 expression and function can similarly occur in primary human endothelial cells (HBMVEC), as well.

In the MDR1 intracellular localization study using confocal microscopy, we have observed similar inductive effects of both HIV-1 and SQV on MDR1 protein levels. The MDR1 (C-219) antibody, which showed clear localization of MDR1 in the cell (green punctate staining) suggested the increased distribution of MDR1 at the cell membrane. A punctate staining pattern for MDR1 on cell membranes has been described previously to be due to their localization at plasma membrane lipid raft like structures, and may also be explained by the presence of MDR1 in vesicles that are closely associated with the plasma membrane [32], [33]. Compared to untreated cells, treatment of SQV and HIV-1 separately induced membrane localization of the MDR1 protein; however, with combined treatment, there was a much higher increase, and is clearly evident in the representative image (Fig. 3). It is possible that both HIV-1 and SQV behave as inducing agents for MDR1 gene and protein expression and combination of these two factors has additive effect on MDR1 function and increased membrane localization. Bauer *et al* (2005) [34], have mentioned that there are three different factors such as location, potency and substrate specificity which render MDR1 a key component of drug regulation in the CNS, and our studies with HIV-1 and SQV exposed HBMVEC demonstrated that all three mechanisms may be involved in increasing drug-efflux function.

The luminal location (on blood side) of MDR1 on BBB makes it very important in the efflux of certain drug substrates back into the blood stream. The substrate specificity of MDR1 on certain HPIs and on nucleoside/nucleotide analogs (NRTIs) causes the drug efflux problem at the BBB to be a formidable barrier. Considering the complex host cellular signaling pathways in response to HIV-1, investigating whether cellular factors or viral factors are responsible for MDR1 function becomes challenging. In the present study, we have observed low endogenous expression and efflux function of MDR1 in primary HBMVEC, although both SQV and HIV-1 act as inducing factors on the efflux function. The calcein-AM and ^3^H-SQV retention studies showed high functional activity of MDR1 and significant inhibition by verapamil alone. There is a possibility that verapamil might block other transporters (MRPs), which are present on the luminal side (blood side), as well. However, considering the gene expression profile, it is evident that the MDR1 activity is much higher than the other transporters. In order to be certain that our present observation is indeed MDR1 mediated, the MDR1 specific blocker, PSC-833 was used and compared with verapamil. In a dose response study with both verapamil and PSC-833, it was evident that by increasing concentration of PSC-833, the MDR1 specific blockade was more prominent. This observation confirmed our present investigation of MDR1 specific induction of drug-efflux by HIV-1 and SQV co-exposure. Furthermore, the MDR1 specific siRNA inhibition study assured the fact that exposure of HIV-1 and/or SQV induces MDR1 specific gene expression and efflux function. Our findings on the effect of virus and drugs on MDR1 efflux function indicates a potential mechanism of HIV-1 drug resistance in the CNS and ultimately resulting in HAART failure for the patients undergoing therapy. Therefore, our study may give insight regarding the pharmacokinetics of other HAART drugs, which are substrates for MDR1, as well.

It is understood that the function of BBB in drug transport regulation in the CNS is a complex mechanism and MDR1 plays an important role in this process [36]. Indeed, MDR1 has been mostly studied in the context of cancer drug resistance [37], [38] and there are only a few other previous publications indicating its crucial role in HAART drug efflux from the CNS [3], [7], [8], [20], [39]. Previous studies on MDR1 did not establish a direct correlation between mRNA and protein expression with MDR1-mediated efflux function [10], [35]; however, our present study indicates that HIV-1 and SQV alone induce expression of this transporter at the transcriptional level only, and concomitant exposure may be needed for significant increases in function. Indeed, functional activity for these transporters is only possible if membrane localization occurs, as shown in our immunofluorescence microscopy studies (Fig. 3). In infected microenvironments, the viral secretary proteins Tat, Nef, Vpu may induce certain host cellular pathways that in turn upregulates MDR1 membrane expression [40], [41]. The HIV-1 Tat-mediated MAP-kinase activation can also upregulate MRP1 expression in mice [31]. Since the MAPK signaling pathways activate multiple downstream second messengers such as ERK1/2, JNK and p38 MAPK pathways, these may also induce MDR1, and/or other transporters, as well [42]. In fact, it is well known that the activated p38 MAPK pathway leads to release of certain inflammatory cytokines from BBB like IL-2, IL-4, IL-6 and TNF-α. These cytokines/chemokines have been implicated in facilitating HIV-1 entry into the CNS through transmigrating via the brain endothelial cell barriers [4], [41], [43]; however, their direct role in activating the BBB endothelial cell drug-efflux mechanisms need to be investigated. Hayashi *et al* (2006) [44] demonstrated that HIV-1 Tat can induce MDR1 expression in mice. Moreover, Tat and HIV-1 gp120 both induces oxidative stress in brain endothelial cells and cause degradation of vascular basement membrane and vascular tight junction even without the presence of intact HIV virion [40], [45], [46]. Although the molecular mechanisms of these regulators are not fully understood, as cell regulatory signals including oxidative stress and inflammatory cytokine secretion, may also contribute to drug efflux activity [11], [23], [47], investigations on the involvement of other host or viral factors in MDR1 regulation within HIV-1 infected foci of the brain needs to be understood. The mechanism of MDR1 based drug regulation at the BBB, where a number of factors may contribute to drug-efflux function, will need to be investigated towards strategies for their specific inhibition. Indeed, our siRNA based study clearly indicated the major involvement of MDR1 in drug-efflux from cells coexposed to HIV-1 and SQV. This implicates that a specific inhibition of MDR1 transporters may provide a clear therapeutic advantage within infected regions of the brain.

## Conclusions

The current study suggests that the MDR1 transporters in the HBMVEC are mainly involved in regulating the entry of HPIs within the CNS. As occurring in HIV-1 infected patients, the concomitant presence of HIV-1-mediated inductive signaling on MDR1 gene expression and the presence of MDR1 substrates such as the HPIs, may act as significant stimulators for the MDR1 mediated drug-efflux function. We observed that HIV-1 exposure alone is not sufficient for increased MDR1 function and co-exposure to HPIs, e.g. saquinavir and/or soluble factors present in HIV-1-infected microenvironments, may be necessary to manifest the increased MDR1 mediated drug-efflux. These data also suggested that the specific inhibition of MDR1 via siRNA or PSC-833 may increase drug entry into the HBMVEC, and thus anti-HIV-1 efficacy within the HIV-1 infected CNS reservoirs. Therefore, targeted suppression of MDR1 (P-gp) expression at the HIV-1 infected reservoirs that overexpress this transporter may introduce novel therapeutic strategies to inhibit drug-efflux from the brain. The utility of siRNA based inhibitory strategies targeted towards the HIV-1-infected CNS foci will also be devoid of the side effects of a generalized inhibition of multiple ABC-transporters or MDR1 inhibition at non-infected sites.
